# Progression and postoperative complications of osteoradionecrosis of the jaw: a 20-year retrospective study of 124 non-nasopharyngeal cancer cases and meta-analysis

**DOI:** 10.1186/s12903-022-02244-9

**Published:** 2022-05-28

**Authors:** Ziqin Kang, Tingting Jin, Xueer Li, Yuepeng Wang, Tianshu Xu, Yan Wang, Zixian Huang, Zhiquan Huang

**Affiliations:** 1 Department of Oral and Maxillofacial Surgery, Sun Yat-Sen Memorial Hospital, Sun Yat-Sen University, 107th Yanjiang Xi Road, Guangzhou, 510120 Guangdong China; 2 Department of Stomatology, Longgang District Central Hospital, Shenzhen, 518116 Guangdong China; 3 Department of Maxillofacial Surgery, The First Affiliated Hospital of Guangdong Pharmaceutical University, Guangzhou, 510080 Guangdong China

**Keywords:** Head and neck cancer, Osteoradionecrosis of the jaw, Postoperative complication, Chemotherapy, Non-nasopharyngeal cancer, Flap reconstruction, Meta-analysis

## Abstract

**Background:**

To assess the contributing risk factors for the progression of, and the postoperative poor prognosis associated with, osteoradionecrosis of jaw (ORNJ) following non-nasopharyngeal cancer treatment in head and neck.

**Methods:**

A retrospective study of 124 non-nasopharyngeal carcinoma patients in head and neck treated at one institution between 2001 and 2020 was conducted. A cumulative meta-analysis was conducted according to PRISMA protocol and the electronic search was performed on the following search engines: PubMed, Embase, and Web of Science. After assessing surgery with jaw lesions as a risk factor for the occurrence of ORNJ, 124 cases were categorized into two groups according to the “BS” classification, after which jaw lesions, chemotherapy, flap reconstruction and onset time of ORNJ were analyzed through the chi-square test and t-test to demonstrate the potential association between them and the progression of ORNJ. Postoperative outcomes of wound healing, occlusal disorders, and nerve injury were statistically analyzed.

**Results:**

With the statistically significant results of the meta-analysis (odds ratio = 3.07, 95% CI: 1.84–5.13, *p* < 0.0001), the chi-square test and t-test were used to validate our hypotheses and identified that surgery with jaw lesions could aggravate the progression and accelerate the appearance of ORNJ. Patients who underwent chemotherapy tended to suffer from severe-to-advanced osteonecrosis but did not shorten the onset time of ORNJ. Flap reconstruction presented obvious advantages in wound healing (*p* < 0.001) and disordered occlusion (*p* < 0.005). The mean onset time of ORNJ in non-nasopharyngeal cancer patients (4.5 years) was less than that in patients with nasopharyngeal cancer (NPC) (6.8 years).

**Conclusions:**

Iatrogenic jaw lesions are evaluated as a significant risk factor in the occurrence and progression of ORNJ in non-nasopharyngeal carcinoma patients who tend to have more severe and earlier osteonecrosis after radiotherapy than NPC patients. Flap reconstruction is a better choice for protecting the remaining bone tissue and reducing postoperative complications of ORNJ.

## Background

Head and neck cancers (HNC) are the seventh most common malignancies worldwide and have been treated effectively by comprehensive sequence therapy, chiefly including surgery, chemotherapy and radiotherapy[1, 2]. Over the past decades, technological advances have transformed radiation therapy (RT) into a precise and effective treatment for cancer patients, and RT has become a crucial actor in cancer management [3]. However, radiotherapy might cause various complications, of which osteoradionecrosis of the jaw (ORNJ) is the most severe and destructive.

The widely accepted definition of ORNJ is that bone lesions and destruction can be observed in the unhealed jaw tissue of the radiation area on imaging for a period of 3–6 months, and recurrence of the primary tumor and new tumors induced by radiation can be excluded [4, 5]. The incidence of osteoradionecrosis is about 4–8% over the past two decades as radiotherapy techniques become more conformal and doses to surrounding tissue decrease [6, 7]. ORNJ can cause emaciation, deformity, and pathological fractures, resulting in decreased quality of life. Patients with ORNJ may have anemia, leukocytosis, hyperproteinemia, and hypercoagulability which might make treatment more challenging [8].

Radiotherapy is the first choice for treatment of nasopharyngeal carcinoma with the promotion of advanced radiotherapy technology [9, 10]. In contrast with NPC, non-nasopharyngeal cancer patients are a noteworthy subset of HNC, and their treatment is more difficult and intractable. However, effective prevention of ORNJ is more significant than effective treatment in both nasopharyngeal and non-nasopharyngeal cancers. ORNJ management is multidisciplinary and can involve multitudinous approaches including conservative treatment, medications, hyperbaric oxygen, curettage of non-vital bone, and more invasive surgical intervention with flap reconstruction. Iatrogenic jaw lesion is defined as irreversible defect or discontinuity of jaw bone caused by surgical procedures following guidelines. Previous studies have displayed numerous high-risk factors for the occurrence of ORNJ, but studies on the risk factors of severe-to-advanced osteonecrosis progression are very rare. This may be due to the lack of consensus on the clear pathogenesis and definition of osteoradionecrosis.

Hence, we performed a retrospective study with 124 non-nasopharyngeal carcinoma patients to evaluate the high-risk factors could aggravate the progression of ORNJ, trying to identified the difference of onset time of ORNJ between non-nasopharyngeal carcinoma and nasopharyngeal carcinoma.

## Methods

### Patients

This retrospective study was performed by the institutional review board of Sun Yat-sen Memorial Hospital at Sun Yat-sen University and the ethics committee. Patients treated at our institution consented in writing for the use of their anonymized data for research purposes. The clinical records and data of ORNJ patients were obtained from the Department of Oral and Maxillofacial Surgery, Sun Yat-sen Memorial Hospital, from September 2001 to October 2020. In total, 124 cases were included in accordance with the selection criteria, which included explicit diagnosis of ORNJ and excluded recurrence of primary tumors. Preoperative examination, treatment, and follow-up data were recorded in patient medical records. The cohort consisted of 124 non-nasopharyngeal carcinoma patients with head and neck cancer who were treated with radiation for primary tumors. Based on the novel clinical classification and staging system, 124 cases were divided into two groups: a mild-to-moderate group (stage 0, stage I) and a severe-to-advanced group (stage II, stage III) [11].

### Meta-analysis

Meta-analysis was performed to evaluate whether jaw lesion increased the risk of osteoradionecrosis after radiotherapy, and the findings provided the basis for our hypothesis that surgery with jaw lesions may promote the progression of ORNJ. In this study, we defined jaw lesion as the loss or discontinuity of jaw bone due to traumatic injuries or jaw surgery. The analysis was conducted following the Preferred Reporting Items for Systematic Reviews and Meta-Analyses (PRISMA) Statement criteria [12]. An electronic search was performed on the following search engines: PubMed, Embase, and Web of Science, without specific filters, from January 1983 to April 2022. The electronic search strategy was conducted by using a combination of the following Medical Subject Headings (MeSH) terms and free text words. PubMed: “Osteoradionecrosis”[Mesh] AND “Prevention and control” [Subheading], “Osteoradionecrosis”[Mesh] AND “Jaw/surgery”[Mesh], “Osteoradionecrosis”[Mesh] AND “Jaw/injuries”[Mesh], “Osteoradionecrosis”[Mesh] AND “Jaw/radiation effects”[Mesh], “Osteoradionecrosis”[Mesh] AND “Head and Neck Neoplasms/surgery”[Mesh], “Osteoradionecrosis”[Mesh] AND “Risk Factors”[Mesh]; Embase: “Osteoradionecrosis”/exp AND “Prevention and control”/exp, “Osteoradionecrosis”/exp AND “Jaw disease”/exp, “Osteoradionecrosis”/exp AND “Oral surgery”/exp, “Osteoradionecrosis”/exp AND “Radiation related phenomena”/exp, “Osteoradionecrosis”/exp AND “Head and neck tumor”/exp, “Osteoradionecrosis”/exp AND “Head and neck surgery”/exp, “Osteoradionecrosis”/exp AND “Risk factor”/exp; Web of Science: “TS = (Osteoradionecrosis) AND TS = (Prevention and control)”, “TS = (Osteoradionecrosis) AND TS = (Jaw surgery)”, “TS = (Osteoradionecrosis) AND TS = (Jaw injuries)”, “TS = (Osteoradionecrosis) AND TS = (Jaw radiation effects)”, “TS = (Osteoradionecrosis) AND TS = (Head and Neck Neoplasms surgery)”, “TS = (Osteoradionecrosis) AND TS = (Risk factor)”. Inclusion criteria: ① full text papers, literature in English language, published after 1983; ② observational clinical studies including randomized clinical trials, prospective studies, cohort and case–control studies; ③ patients underwent radiotherapy and surgery causing jaw lesions with HNC. Exclusion criteria: ① case reports, reviews, conference literature, cross-sectional studies; ② studies without distinct definition of ORNJ; ③ jaw lesion was caused by tooth extraction and occurred after the diagnosis of ORNJ. A cumulative meta-analysis was performed with a random effects model in accordance with the inverse variance method. The odds ratio (OR) of the risk of ORN occurrence was calculated. The results of the meta-analysis were presented in a forest plot graph. The software RevMan version 5.2 was used to perform the statistical analysis.

### Therapeutic category

The study generally included four treatments for ORNJ. In the conservative treatment group, 11 patients were treated with simple symptomatic treatment, mainly divided into three aspects: hyperbaric oxygen therapy; anti-inflammatory, antifibrosis and analgesic medication; and cell growth factor therapy. For surgical treatments, 43 patients underwent simple curettage with the excision of the fistula; 23 patients with partial jaw excision were divided into marginal jaw resection and segmental jaw resection on the basis of the depth of infiltration of necrotic bone and patients’ overall condition after stabilizing inflammation, intermaxillary traction was necessary at the same period; in total, 47 patients underwent vascularized tissue flap reconstruction combined with resection of necrotic tissue.

### Evaluation of postoperative effects

One of the objectives of this study was to evaluate the effect of different surgical treatments. Most patients had a good recovery after effective surgical treatment, but several common complications also affected them. Complication categories included: wound healing, occlusal disorders and nerve injury. Follow-up was from the time the patient of ORNJ underwent surgery treatment to the present, and the evaluation criteria of the abovementioned complications were as follows: ① wounds with inflammatory reactions such as redness, hematoma, effusion, and even suppuration; ② mandibular deviation, chewing weakness and temporomandibular joint popping and pain; ③ deflection of angle of mouth, drum cheek weakness, involuntary drooling and numbness of lips, teeth and tongue.

### Evaluation of chemotherapy and jaw lesions in the progression of ORNJ

After assessing surgery with jaw lesions as a risk factor for the appearance of ORN by meta-analysis, this study decided to evaluate whether two factors, chemotherapy and jaw lesions, could promote the progression of ORNJ based on the “BS” classification among 124 non-nasopharyngeal carcinoma patients. A total of 58 patients underwent surgery involving the jaw with primary tumor resection, including paramedian or median mandibular osteotomy, marginal mandibulectomy or maxillectomy and segmental mandibulectomy. Then, we further examined whether jaw lesions with flap reconstruction before radiotherapy could slow the progression of ORNJ. In addition, 46 patients were divided into the chemoradiotherapy group, and 78 patients only underwent radiotherapy. However, patients’ specific chemotherapy and radiotherapy regimens cannot be fully tracked due to the long time and their treatments in different institutions. The average time elapsed between the end of radiotherapy and clinical diagnosis of ORNJ was then compared. Furthermore, to compare the onset time of ORNJ with nasopharyngeal cancer and non-nasopharyngeal cancer in HNC patients, another 180 cases of nasopharyngeal cancer were recruited from a previous study of osteoradionecrosis by our team [8].

### Statistical analysis

A total of 124 patients’ general demographic data were analyzed with descriptive statistical analysis, including sex, age, primary tumor site, classification, onset time of ORNJ and postoperative complications. After evaluating jaw lesions as a risk factor on the occurrence of ORNJ by meta-analysis, Pearson’s chi-square tests and Student’s t tests were used for bivariate analysis. Differences with *p* < 0.05 were considered statistically significant. All analyses were performed using the Statistical Package for Social Sciences for Windows software (version 25.0, IBM Corp).

## Results

### Patients and treatments related characteristics

As displayed in Table 1, in 124 non-nasopharyngeal carcinoma patients, there were more than twice as many male patients as female patients, and the mean age was 57.6 (10.1) years (range 32–83). Most cases of primary tumors were oral carcinoma (89%), and the average onset time of ORNJ was 4.5 years (range 1 month-32 years). Table 2 shows 124 cases’ BS classification; 6 (5%) of 124 ORNJ patients were grouped into stage 0, and 62 (50%) were grouped into stage I. Forty-four patients (35%) were grouped into stage II, and 12 (10%) out of 124 patients with ORNJ presented with advanced pathological fracture (stage III). A total of 47 patients underwent vascularized tissue flap reconstruction with stage II and III disease, including 33 cases that were repaired with free flaps and 14 that were repaired with pedicled flaps. The free flap reconstruction comprised 30 cases of fibular osteomyocutaneous flaps, one case of anterolateral femoral free flap, one case of forearm flap and one case of iliac osteomusculocutaneous flap. In this study, 3 cases of skin flaps failed to repair the lesion and a second operation was performed, including 2 cases of fibular osteomyocutaneous flaps and one case of pectoralis major myocutaneous flap.

**Table 1:** Demographic and clinical characteristics of study patients according to ORNJ

Variable	
No. of patients	124
Mean (SD) range (years)	57.6 (10.1), 32–83
Sex (male/female)	89/35
Chemoradiotherapy/ Radiotherapy only	46/78
Type of primary tumor:	
Tongue carcinoma	41 (33.1%)
Gingival carcinoma	10 (8.1%)
Buccal carcinoma	9 (7.3%)
Mouth floor carcinoma	11 (8.9%)
Oropharyngeal carcinoma	23 (18.5%)
Hard palate carcinoma	3 (2.4%)
Salivary gland carcinoma	6 (4.8%)
Carcinoma of jaw	3 (2.4%)
Maxillary sinus carcinoma	4 (3.2%)
Other	14 (11.3%)
Mean (SD) range time from end of radiotherapy to development of osteonecrosis of the jaws	4.5 (5.1) years, 1 months-32 years

*ORNJ* osteoradionecrosis of jaw; *SD* standard deviation; *No.* number

**Table 2:** Bone (B) and soft tissue (S) classification, and stage of osteonecrosis of the mandible

Variable	Stage (n)
Bony destruction:	
BO (no bony exposure, only imaging)	0 (6)
B1 (exposed bone, area < 2.0 cm)	B0S0
B2 (exposed bone, ≥ 2 cm)	I (62)
B3 (pathological fracture)	B1S0,B1S1,B1S2
Soft tissue injury:	II (44)
SO (no soft tissue damage)	B2S0,B2S1,B2S2
S1 (mucosal or skin damage)	III (12)
S2 (mucosal and skin damage)	B3S0,B3S1,B3S2

### Meta-analysis

**Fig. 1:**
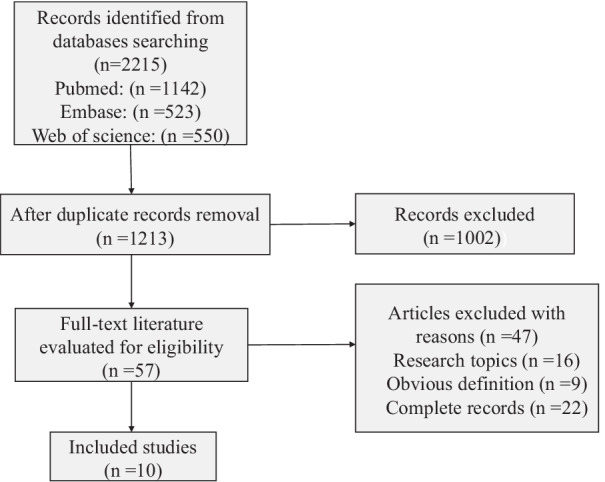
The screening process was conducted according to the PRISMA flow-diagram. Ten articles were finally included in the meta-analysis

**Fig. 2:**
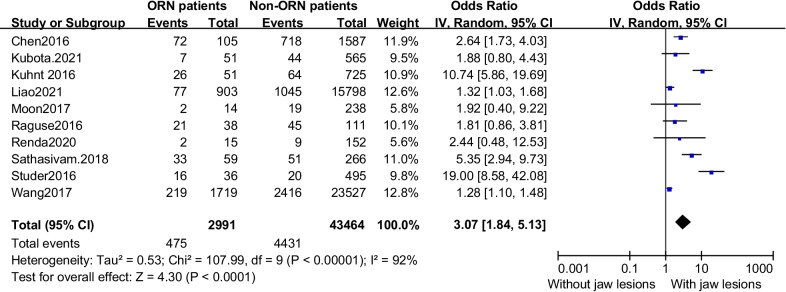
The forest plot graph shows the OD of risk of developing ORNJ in irradiated patients undergoing surgery with jaw lesions. Abbreviations: CI, Confidence Interval; I^2^, Higgins’ Hindex; IV, Inverse Variance

The results of the search and papers selection of the meta-analysis are shown in Fig. 1. The electronic search provided 2215 records (PubMed: 1142 papers, Embase: 523 papers, Web of Science: 550). Eventually, ten articles conformed to the criteria [13–22]. General information regarding patients who underwent jaw surgery is presented in Table 3. In total, 4906 patients underwent surgery with jaw lesions out of 46,455 samples overall who suffered from HNC. Among these patients, 475 developed ORNJ, and the results of statistical analysis are displayed in Fig. 2. The forest plot graph showed the presence of a high rate of heterogeneity between the studies (I^2^ = 92%), and jaw lesion was a high-risk factor for the occurrence of osteonecrosis (OR = 3.07, 95% CI: 1.84–5.13, *p* < 0.0001).

**Table 3:** Characteristics of included patients: among 4906 patients who received surgery with jaw lesions, 475 ORN were diagnosed

Study	No. of Patients	Mean time of follow-up (months)	Patients receiving surgery with jaw lesions	ORN patients with jaw lesions
Wang et al. [13]	25,246	19.1	2635	219
Studer et al. [14]	531	38	36	16
Sathasivam et al. [15]	325	44.1	84	33
Renda et al. [16]	167	50.4	11	2
Raguse et al. [17]	149	41	66	21
Moon et al. [18]	252	25	21	2
Liao et al. [19]	16,701	75	1122	77
Kuhnt et al. [20]	776	–	90	26
Kubota et al. [21]	616	40	51	7
Chen et al. [22]	1692	–	790	72

*ORN* osteoradionecrosis; *No.* number

### Postoperative complications of ORNJ

In Table 4, a total of 113 patients (90%) underwent surgical treatments after the diagnosis of ORNJ, but only 90 patients’ postoperative complications, including wound healing, occlusal disorders and nerve injury, were statistically analyzed because the other patients were lost to follow-up. A statistically significant difference was demonstrated among the three treatment groups involving wound healing (*p* < 0.001) and occlusal disorders (*p* = 0.022). Flap reconstruction after partial resection of the mandible can greatly reduce the incidence of postoperative complications such as poor wound healing and occlusal disorders. However, there was no significant difference in the postoperative incidence of nerve injury between the three surgical treatments (*p* = 0.152).

**Table 4:** Evaluation of poor prognosis for different surgical approaches

Postoperative complications	Treatment	Poor	Favorable	*P* value
Wound healing	Flap reconstruction (n = 40)	6 (15%)	34 (85%)	< 0.001^b^
	Partial mandibulectomy (n = 17)	8 (47%)	9 (53%)	
	Curettage of non-vital bone (n = 33)	19 (57.6%)	14 (42.4%)	
Disordered occlusion	Flap reconstruction (n = 40)	8 (20%)	32 (80%)	< 0.005^b^
	Partial mandibulectomy (n = 17)	11 (64.7%)	6 (35.3%)	
	Curettage of non-vital bone (n = 33)	13 (39.4%)	20 (60.6%)	
Nerve injury	Flap reconstruction (n = 40)	12 (30.0%)	28 (70.0%)	0.152^b^
	Partial mandibulectomy (n = 17)	9 (52.9%)	8 (47.1%)	
	Curettage of non-vital bone (n = 33)	16 (48.4%)	17 (51.6%)	

^b^Pearson’s chi-square test

### Risk factors promote the progression of ORNJ

Overall treatments of 124 cases were obtained through a detailed examination of patient medical records for radiographic images and reports and histopathology reports. Before radiotherapy, 58 patients underwent primary tumor resection with jaw lesions, and 37 patients did not have jaw lesions. In addition, 29 patients underwent radiotherapy only (*p* = 0.018), indicating that radiotherapy and surgery with jaw lesions may exacerbate the progression of severe advanced osteonecrosis. Then, three different surgical procedures with jaw lesions were analyzed, and there was no significant difference between paramedian or median mandibular osteotomy, marginal mandibulectomy or maxillectomy and segmental mandibulectomy (*p* = 0.133). Among the 58 patients with jaw lesions, the flap reconstruction groups (43.8%) had significantly slower progression of osteonecrosis than patients who were not repaired with flaps (*p* = 0.011). Similarly, the paramedian or median mandibular osteotomy group and marginal mandibulectomy or maxillectomy group underwent chi-square tests according to the presence or absence of flap reconstruction, and the *p* values were 1.000 and 0.014, respectively. Bivariate statistical analysis of chemoradiotherapy and jaw lesions was conducted, which further indicated that both single chemoradiotherapy and chemoradiotherapy combined with jaw lesions can promote the progression of osteonecrosis, as shown in Table 5.

**Table 5:** Effects of jaw lesions and chemotherapy for progression of ORNJ and the onset time compared with different variables

Variables	All cases in study (n = 124)	Patients with BS stage 0, I	Patients with BS stage II, III	*P* value
Overall treatment				0.018^b^
Radiotherapy only	29 (23.4%)	19 (65.5%)	10 (34.5%)	
Radiotherapy and surgery with jaw lesions	58 (46.8%)	24 (41.4%)	34 (58.6%)	
Radiotherapy and surgery without jaw lesions	37 (29.8%)	25 (67.6%)	12 (32.4%)	
Surgery involving the jaw				0.133^b^
Paramedian or median mandibular osteotomy	20 (16.1%)	5 (25%)	15 (75.0%)	
Marginal mandibulectomy or maxillectomy	30 (24.2%)	14 (46.7%)	16 (53.3%)	
Segmental mandibulectomy	8 (6.5%)	5 (62.5%)	3 (37.5%)	
Surgery with flap reconstruction	32 (25.8%)	18 (56.2%)	14 (43.8%)	0.011^b^
Surgery without flap reconstruction	26 (21.0%)	6 (23.1%)	20 (76.9%)	
Different jaw lesions with flap reconstruction				0.115^b^
Paramedian or median mandibular osteotomy	8 (6.4%)	2 (25%)	6 (75%)	
Marginal mandibulectomy or maxillectomy	16 (12.9%)	11 (68.8%)	5 (31.2%)	
Segmental mandibulectomy	8 (6.4%)	5 (62.5%)	3 (37.5)	
Paramedian or median mandibular osteotomy with flap reconstruction	8 (6.4%)	2 (25%)	6 (75%)	1.000^b^
Paramedian or median mandibular osteotomy without flap reconstruction	12 (9.8%)	3 (25%)	9 (75%)	
Marginal mandibulectomy or maxillectomy with flap reconstruction	16 (12.9%)	11 (68.8%)	5 (31.2%)	0.014^b^
Marginal mandibulectomy or maxillectomy without flap reconstruction	14 (11.3%)	3 (21.4%)	11 (78.6%)	
Chemoradiotherapy and jaw lesions				
Chemoradiotherapy	46 (37.0%)	17 (37.0%)	29 (63.0%)	0.004^b^
Radiotherapy only	78 (63.0%)	51 (65.4%)	27 (34.6%)	
Chemoradiotherapy with jaw lesions	16 (13.0%)	1 (6.2%)	15 (93.8%)	< 0.001^b^
Radiotherapy only with jaw lesions	42 (33.9%)	23 (54.8%)	19 (45.2%)	
Chemoradiotherapy without jaw lesions	30 (24.2%)	16 (53.3%)	14 (46.7%)	0.036^b^
Radiotherapy only without jaw lesions	36 (29.0%)	28 (77.8%)	8 (22.2%)	
Chemoradiotherapy with jaw lesions	16 (13.0%)	1 (6.2%)	15 (93.8%)	0.002^b^
Chemoradiotherapy without jaw lesions	30 (24.2%)	16 (53.3%)	14 (46.7%)	
Radiotherapy only with jaw lesions	42 (33.9%)	23 (54.8%)	19 (45.2%)	0.033^b^
Radiotherapy only without jaw lesions	36 (29.0%)	28 (77.8%)	8 (22.2%)	
Onset time of ORN, mean years ($\pm$ SD)				
Surgery with jaw lesions (n = 58)	3.3 (3.8)			0.008^a^
Surgery without jaw lesions (n = 66)	5.7 (5.7)			
Jaw lesions with flap reconstruction (n = 32)	2.9 (3.3)			0.484^a^
Jaw lesions without flap reconstruction (n = 26)	3.6 (4.3)			
Chemoradiotherapy (n = 46)	4.3 (4.2)			0.637^a^
Radiotherapy only (n = 78)	4.8 (5.5)			
Chemoradiotherapy with jaw lesions (n = 16)	2.4 (3.6)			0.331^a^
Radiotherapy only with jaw lesions (n = 42)	3.5 (3.8)			
Chemoradiotherapy without jaw lesions (n = 30)	5.0 (4.3)			0.396^a^
Radiotherapy only without jaw lesions (n = 36)	6.3 (6.7)			
Radiotherapy only with jaw lesions (n = 42)	3.5 (3.8)			0.025^a^
Radiotherapy only without jaw lesions (n = 36)	6.3 (6.7)			
Chemoradiotherapy with jaw lesions (n = 16)	2.4 (3.6)			0.041^a^
Chemoradiotherapy without jaw lesions (n = 30)	5.0 (4.3)			
Nasopharyngeal carcinoma patients (n = 180)	6.8 (4.8)			< 0.001^a^
Non-nasopharyngeal carcinoma patients (n = 125)	4.5 (5.1)			

^a^Student’s t test

^b^Pearson’s chi-square test

Among the above variables that were statistically significant in promoting the progression of osteonecrosis, Student’s t tests were adopted to evaluate the onset time of ORNJ. Statistical results showed that jaw lesions not only aggravated the progression of osteonecrosis but also shortened the onset time of ORNJ. In contrast, jaw lesions with flap reconstruction and chemoradiotherapy did not affect the onset time of ORNJ (*p* > 0.05). Furthermore, after comparing 180 nasopharyngeal cancer patients (6.8 years) with 124 non-nasopharyngeal cancer patients (4.5 years), the mean onset time of osteonecrosis in the former was significantly longer than that in the latter (*p* < 0.001).

## Discussion

Osteoradionecrosis of the jaws (ORNJ) is an insidious complication of radiotherapy for head and neck carcinomas. Osteoradionecrosis induced by radiotherapy for nasopharyngeal carcinoma has been widely and systematically studied. However, there is little literature regarding the progression and prognosis of osteoradionecrosis caused by treatment of non-nasopharyngeal cancer.

Compared with nasopharyngeal cancer, which is sensitive to radiotherapy, the treatment of non-nasopharyngeal cancer patients is more complicated and intractable. As the main part of non-nasopharyngeal carcinoma, OSCC represents a specific entity in terms of its management and therapeutic outcomes [23]. The incidence of jaw lesions in primary tumor resection surgery is higher than that in other non-nasopharyngeal cancers, such as thyroid cancer, laryngeal cancer, and lymphoma in HNC. Regarding the pathogenesis of ORN, many classical theories have been reported in the literature. Marx [4] published the famed hypoxic-hypocellular-hypovascular theory and indicated that trauma is only one mechanism of tissue breakdown leading to complications. In 2012, S L Wang et al. [24] suggested that microvessel damage may play a key role in the occurrence and development of ORN. Based on the above theory, jaw lesions and chemotherapy drugs cause damage to blood circulation and microvessels.

Results of this research showed that 58 patients experienced jaw lesions owing to extensive resection of the primary tumor site as a surgical approach to remove the tumor completely, which helps to reduce tumor recurrence. The influence of developing ORNJ is probably caused by surgical interruption of the blood circulation of the jaw [25]. On the other hand, maintaining the integrity of the periosteum as much as possible is a critical factor, and the decrease in the number of cells was considered to be connected with injury to the bone marrow and the periosteum and from the decrease in the number of osteoblasts [4, 26]. New bone tissue cannot be regenerated, and this in combination with the damage and embolization of blood supply, the risk of aggravating ORNJ is greatly increased. In this study, jaw lesions were divided into three groups according to surgical procedures: simple mandibular osteotomy, marginal jaw resection and segmental mandibulectomy. The statistical analysis shows that the progression of osteonecrosis was not influenced by different surgical approaches (*p* = 0.133). Although we cannot evaluate the effects of jaw damage caused by these three diverse surgical procedures on the progression of ORN, what they all have in common is performing paramedian or median mandibular osteotomy. Therefore, median or paramedian mandibular osteotomy due to iatrogenic jaw lesions is assessed as a vital risk factor in the progression of ORNJ in non-nasopharyngeal carcinoma patients.

The findings of this study about chemoradiotherapy are that it can aggravate the progression of osteonecrosis but does not accelerate the occurrence. Admittedly, chemotherapy drugs have toxic effects on normal vascular endothelial cells, causing vascular inflammation that may lead to local occlusion and even necrosis. However, some previous literature has reported that chemotherapy is a protective factor for the occurrence of ORNJ [13, 18]. Meanwhile, this retrospective study lack specific chemotherapy regimens in patients of ORNJ as a limiting factor. Hereto, we reserve our own opinions and will conduct a more rigorous and careful design in the future.

With the development of microsurgical techniques, pedicle flaps and free flaps have been widely used in the treatment of HNC. In our study, Table 4 indicates that flap repair has obvious advantages in reducing the occurrence of poor wound healing and occlusal disorders. After all, well-vascularized tissue flap reconstruction can remedy the damaged or interrupted blood circulation of the jaw and act as a protective barrier for irradiation of the remaining bone tissue. For patients with dentition defects, intermaxillary traction is obligatory but not enough to improve the accuracy of jaw reconstruction in ORNJ patients [27, 28]. Compared with ORNJ, the treatment of bisphosphonate-related osteonecrosis of the jaw (BRONJ) is less extensive and effective [29]. Surgical debridement produces more bone necrosis and it’s unavailing to cover the exposed areas with tissue flaps due to the entire skeleton is being treated with the bisphosphonate. Hyperbaric oxygen therapy and antibiotics also have little effects. Prevention is the only currently possible therapeutic approach to the management of BRONJ which is consistent with ORNJ. Generally, for ORNJ patients in stage II or III, flap reconstruction with dental implants treatment is still recommended as a priority, and dentists should advise patients who underwent partial mandibulectomy and curettage to repair defective dentition in a timely manner.

Oral health is the bridge to well-being, abundant clinical evidences have reported periodontal disease negatively affects the whole body, and it has a close association with diabetes and cardiovascular diseases [30]. Consequently it is closely correlative that poor oral health may promote the progression of ORNJ. Overall, iatrogenic jaw lesion is a high-risk factor for the progression of osteonecrosis, and oral surgeons should schedule different frequencies of follow-up and readmission for patients, depending on the patient’s previous treatment, including surgery with jaw lesion and radiation therapy. Radiologists should also pay extra attention to HNC patients with jaw lesions, especially who have underwent paramedian or median mandibular osteotomy, and strive to achieve precision radiation therapy and help patients understand the association between radiation and ORNJ, educational audiovisual tools may be a good choice [31]. Achieving early prevention and intervention for ORNJ is dependent on the surgeon’s sense of responsibility and the patient’s consciousness and active cooperation.

Recently, there has been a surging interest in the development of clinical prognostic models of OSCC, particularly in nomograms which are their graphic representation [32, 33]. It is of clinical significance to conduct similar study to predict OSCC patient outcomes after RT for the onset time and progression of ORNJ by incorporating multiple variables including tumor-related factors, bone invasion, chemotherapy, iatrogenic jaw lesions, radiotherapy, diabetes and other high risk factors, which is very important for oral health education, treatment planning, follow-up, and postoperative risk assessment in OSCC patients after RT. Prospective cohort studies will be performed for predictive modeling as the future perspectives of our study.

## Conclusions

In summary, the study findings indicated iatrogenic jaw lesions are assessed as a risk factor in the occurrence and progression of ORNJ in non-nasopharyngeal cancers. Flap reconstruction can retard the progression of osteonecrosis and is more suitable for repairing severe and advanced ORNJ. But the retrospective nature of this study was a limiting factor as was the reliance on obtaining data from medical records completed by multitudinous doctors and lacked specific records of chemotherapy regimens, radiation doses and radiation approaches. All in all, it is indispensable to choose the most suitable personalized treatment, follow-up strategy and perform oral health education for each radiotherapy patient in HNC.

## Data Availability

The datasets generated and analyzed during the current study are not publicly available due to (ownership of data) but are available from the corresponding author on reasonable request.

## Abbreviations

ORNJ: Osteoradionecrosis of jaw
HNC: Head and neck cancer
NPC: Nasopharyngeal carcinoma
SD: Standard deviation
No.: Number
ORN: Osteoradionecrosis
CI: Confidence Interval
I^2^: Higgins’ Hindex
IV: Inverse Variance
IMRT: Intensity-modulated radiotherapy
OR: Odds ratio
OSCC: Oral cavity squamous cell carcinoma
RT: Radiotherapy
BRONJ: Bisphosphonate- related osteonecrosis of the jaw
